# Discriminative Analysis of Migraine without Aura: Using Functional and Structural MRI with a Multi-Feature Classification Approach

**DOI:** 10.1371/journal.pone.0163875

**Published:** 2016-09-30

**Authors:** Qiongmin Zhang, Qizhu Wu, Junran Zhang, Ling He, Jiangtao Huang, Jiang Zhang, Hua Huang, Qiyong Gong

**Affiliations:** 1 Department of Medical Information Engineering, School of Electrical Engineering and Information, Sichuan University, Chengdu, Sichuan, China; 2 Monash Biomedical Imaging, Monash University, Melbourne, Victoria, Australia; 3 Huaxi MR Research Center (HMRRC), Department of Radiology, West China Hospital of Sichuan University, Chengdu, Sichuan, China; 4 Computer and Information Engineering School, Guangxi Teachers Educational University, Nanning, Guangxi, China; Yale University, UNITED STATES

## Abstract

Magnetic resonance imaging (MRI) is by nature a multi-modality technique that provides complementary information about different aspects of diseases. So far no attempts have been reported to assess the potential of multi-modal MRI in discriminating individuals with and without migraine, so in this study, we proposed a classification approach to examine whether or not the integration of multiple MRI features could improve the classification performance between migraine patients without aura (MWoA) and healthy controls. Twenty-one MWoA patients and 28 healthy controls participated in this study. Resting-state functional MRI data was acquired to derive three functional measures: the amplitude of low-frequency fluctuations, regional homogeneity and regional functional correlation strength; and structural MRI data was obtained to measure the regional gray matter volume. For each measure, the values of 116 pre-defined regions of interest were extracted as classification features. Features were first selected and combined by a multi-kernel strategy; then a support vector machine classifier was trained to distinguish the subjects at individual level. The performance of the classifier was evaluated using a leave-one-out cross-validation method, and the final classification accuracy obtained was 83.67% (with a sensitivity of 92.86% and a specificity of 71.43%). The anterior cingulate cortex, prefrontal cortex, orbitofrontal cortex and the insula contributed the most discriminative features. In general, our proposed framework shows a promising classification capability for MWoA by integrating information from multiple MRI features.

## Introduction

Migraine is a form of primary neurovascular disorder characterized by episodic headache [1]. According to a survey by the World Health Organization, migraine ranks as the third most prevalent disorder, and affected nearly 15% of the whole population. More than 90% of sufferers are unable to work or function normally during their migraine attacks [2]. Moreover, people who undergo migraine may have an increased risk of ischemic stroke, unstable angina, or affective disorders [3–5]. Based on whether the headaches are accompanied by an early symptom that called aura, migraine is divided into two major subtypes: migraine without aura (MWoA) and migraine with aura. It is worth to note that two thirds of migraine patients suffer from MWoA [6], hence the early diagnosis and appropriate treatment of MWoA is imperative. Since MWoA has no clear prodrome, and the symptoms are hard to evaluate and may change from one attack to the next, it's not always easy to exclude other possible causes of headache and achieve an accurate diagnosis of MWoA using traditional methods (e.g. symptoms analysis, medical tests). Thus, there has been substantial interest in identifying objective biomarkers and developing automated methods that with the potential to assist the diagnosis of migraine.

In recent years, studies using magnetic resonance imaging (MRI) have greatly advanced our understanding to the neural mechanisms underlying migraine. Both structural and functional brain alterations in migraine have been revealed by MRI techniques. Specifically, studies using structural MRI (sMRI), especially high-resolution T1-weighted imaging, have demonstrated that migraine is linked with gray matter (GM) changes in the inferior parietal lobule [7], hippocampus [8], inferior frontal cortex [9], motor/premotor and the prefrontal cortex [10]; a recent study on migraineurs [11] also revealed changes in the regional cortical thickness, cortical surface area, and volume in several brain areas including the parahippocampal gyrus, anterior cingulate cortex and the medial orbital frontal gyrus. Meanwhile, by employing resting-state functional MRI (rs-fMRI), a number of studies have demonstrated that migraine was associated with functional brain alterations measured by various indices, including the amplitude of low-frequency fluctuations (ALFF), regional homogeneity (ReHo), as well as functional connectivity. For example, compared with the healthy controls, migraineurs showed significant ALFF changes in the anterior cingulate cortex and prefrontal cortex [12], and ReHo changes in the prefrontal cortex, orbitofrontal cortex [13], insula [14], and cuneus [15]. Altered functional connectivity in migraineurs was identified between the dorsolateral prefrontal cortex and the dorsal anterior cingulate cortex [16], between amygdala and insula [17], and between the prefrontal and temporal regions that were within the default mode network [18].

Despite identification of the aforementioned functional and morphological brain alterations, there is very few exploration on the possibility of utilizing the MRI findings to assist diagnosis of migraine patients. One important reason is that most of these findings were obtained by applying mass-univariate analysis approaches to detect group differences [19], however, for neuroimaging to be useful in a clinical setting, one must be able to provide predictions at the individual level. In the past several years, the application of machine learning techniques to neuroimaging data analysis has made promising progress in brain disease identification [20, 21]. Compared to the group level analyses, machine learning techniques allow inference at the single-subject level, and moreover, they are sensitive to subtle and spatially distributed differences in the brain that might be undetectable in group level comparisons. Recently, a growing number of studies have used machine learning methods to examine a range of psychiatric and neurological conditions, such as Alzheimer’s disease [22], autism [23], social anxiety disorder [24], depression [25], and schizophrenia [26].

In the application of machine learning techniques, one can either use features derived from single-modality MRI data or even a single measure, or include multi-modality features. The advantage of the latter way is that different MRI modalities/measures usually provide complementary pathological information. Aside the obvious distinction between sMRI and functional MRI, the above-mentioned three indices derived from rs-fMRI are also mutual-complementary. In detail, both ALFF and ReHo measure the regional spontaneous neural activity, but ALFF reflects the amplitude [27] while ReHo indicates the functional synchronization of neural units that are spatially close to each other [28]. Functional connectivity, i.e., the connectivity between separate brain regions, provides functional information at the level of brain networks [29]. By taking them together, one can achieve a more comprehensive understanding of the brain function from segregation to integration [30]. The advantage of combining multi-type features over single-type features was also verified in recent studies by showing improved classification performance in various diseases, including ADHD [31], Alzheimer’s disease [32–34], Parkinson’s disease [35], and schizophrenia [36]. Nonetheless, very few studies have tried the multi-type features combination approach in migraine discrimination, and the capability of machine learning techniques for this condition is not yet known.

With the hypothesis that integration of multi-type features in a proper way could improve the classification performance compared with single-type feature approaches, in the current study, we proposed a novel framework that employs multi-kernel support vector machine (SVM) to combine ALFF, ReHo, regional functional correlation strength (RFCS) and GM features. We examined whether this framework works better than single-type feature approaches in differentiating MWoA patients from healthy controls (HC).

## Materials and Methods

### Subjects

Twenty-one migraine patients without aura and twenty-eight healthy controls participated in this study. The age and gender differences between the two groups were tested using two-sample t-test and χ^2^ test respectively, and no significant difference was observed (*p*>0.05). The diagnosis of MWoA was made by neurologic practitioners according to the criteria from the Second Edition of the International Classification of Headache Disorders (ICHD-II) [37]. All the patients were right-handed and aged between 18 and 45 years. They had to be off analgesic drugs for at least 2 weeks, not in preventive treatment and had not used any other drugs for at least 1 month prior to the study. All patients were free from migraine attack during a follow-up for at least 72 hours prior to the brain scan, and 48 hours after the scan. Exclusion criteria included: patients with chronic migraine or other chronic or current pain disorders, subjects with a history of mental diseases or other neurological disorders besides migraine, pregnant females, subjects with MRI contraindications, or with structural abnormalities in brain found by computer tomography or conventional MRI scanning. The Local Ethics Committee of the West China Hospital of Sichuan University approved this study and all subjects have given written informed consents prior to the participation. Table 1 lists the demographic and clinical data of the 49 subjects.

**Table 1:** Demographic and clinical characteristics of the 49 participants.

	MWoA (*n* = 21)	HC (*n* = 28)	*T-*value	χ^2^ value	*p*-value
**Sex (male/female)**	5/16	13/15	—	2.642	0.104^a^
**Age (mean ± SD, y)**	27.52 ± 8.15	29.18 ± 6.96	-0.766	—	0.448^b^
**Education (mean ± SD, y)**	15.05 ± 4.14	16.36 ± 2.87	-1.308	—	0.197^b^
**24-HAMD (mean ± SD)**	6.48 ± 6.46	2.46 ± 2.17	2.735	—	0.012^b^
**14-HAMA (mean ± SD)**	4.38 ±5.50	1.46 ± 1.43	2.371	—	0.027^b^

SD = standard deviation; y = year; HAMD = Hamilton Depression Scale; HAMA = Hamilton Anxiety Scale; MWoA = migraine without aura; HC = healthy controls.

^a^ The p value was obtained by χ^2^ test.

^b^ The p values were obtained by two-sample t-test.

### Image acquisition

All data were acquired using a 3.0 Tesla MRI system (Trio Tim, Siemens, Erlangen, Germany). Foam paddings and headphones were used to limit head movement and reduce scanner noise for the subjects. During the data acquisition, all participants were instructed to keep their eyes closed but not fall asleep, relax their minds and keep as still as possible. A three-dimensional magnetization-prepared rapid gradient echo sequence was used to collect T1-weighted structural images, with the following parameters: repetition time/echo time (TR/TE) = 1900/2.26 ms, flip angle = 9°, slice thickness/gap = 1/0 mm, field of view (FOV) = 256 × 256 mm^2^, matrix = 256 × 256, voxel size = 1 × 1 × 1 mm^3^. The rs-fMRI data were collected using an echo planar imaging (EPI) sequence: TR/TE = 2000/30 ms, flip angle = 90°, slice thickness/gap = 5/0 mm, FOV = 240 × 240 mm^2^, matrix = 64 × 64, voxel size = 3.75 × 3.75 × 5 mm^3^.

### Data preprocessing

Structural images were preprocessed using the Statistical Parametric Mapping software (SPM8, http://www.fil.ion.ucl.ac.uk/spm). Images were first segmented into GM, white matter (WM) and cerebrospinal fluid partitions, then the GM and WM partitions were utilized to create a study-specific template using the diffeomorphic anatomical registration through exponentiated lie algebra (DARTEL) algorithm [38]. The individual GM images were warped to this template, and then modulated and resliced with the resolution remained. Finally, a Gaussian kernel with a full-width at half-maximum (FWHM) of 8 mm was used to smooth all the GM images.

Resting-state functional images were preprocessed using SPM8 and the Data Processing Assistant for Resting-State fMRI (DPARSF, http://rfmri.org/DPARSF) toolbox. The first 10 EPI volumes were discarded to avoid the magnetic saturation effects and ensure all participants adapted to the scanning environment. The remaining volumes first underwent slices timing correction, and then realigned to the first volume to correct for susceptibility-by-movement interaction. None of the subjects’ head in this study has a movement that exceeds 2 mm displacement and 2° of rotation in any direction. The realigned scans were further spatially normalized to the Montreal Neurological Institute template and resliced to 3 mm isotropic voxels. Next, band-pass filtering (0.01 Hz—0.08 Hz) was performed on the time series of each voxel to reduce the effect of low-frequency drifts and high-frequency physiological noise [39]. Then the ALFF, ReHo and RFCS were calculated as described below.

### Feature extraction

ALFF is an effective indicator of regional intrinsic or spontaneous neuronal activity in the brain [40]. In the calculation of ALFF, the normalized and resliced images were firstly smoothed using a 4 mm FWHM Gaussian kernel. Then the ALFF, within the frequency band 0.01 Hz—0.08 Hz, was calculated for each voxel using the Resting-State fMRI Data Analysis Toolkit (REST, http://rest.restfmri.net). To reduce the global effects of variability across participants, for a certain subject, the ALFF of each voxel was divided by the global mean ALFF value. The individual ALFF maps were then partitioned into 116 regions of interest (ROIs) based on the automated anatomical labeling (AAL) atlas [41], and the 116 regional mean ALFF values were extracted as features for this subject.

ReHo, which measures the functional synchronization of a given voxel with its nearest neighbors, was also calculated using the REST software. For each normalized and resliced image, the measured cluster was set as 27 voxels [28]. Similar to ALFF processing, the ReHo of each voxel was also divided by the global mean ReHo value. After smoothing with a 4 mm FWHM Gaussian kernel, a ReHo map was obtained for each subject and partitioned into 116 ROIs using the AAL atlas. The ReHo features of one subject were consisted of the 116 regional mean ReHo values.

The RFCS measures the average correlation extent of a given brain region compared with all other regions [32]. To compute resting-state functional connectivity, we regressed out the spurious effects of nuisance covariates [42]. The individual volume was first partitioned into 116 ROIs using the AAL atlas, and the mean time series of each region was then extracted by averaging the time series within that region. We obtained a 116×116 correlation matrix for each subject by calculating the Pearson correlation coefficients between all possible pairs of regions. Then, the RFCS was calculated using a method that has been described in previous studies [43]. The *i*-th RFCS was defined as:

$$S(i)=\frac{1}{N-1}\sum_{j\neq i}\left|R_{ij}\right|$$ (1)

where R_*ij*_ is the correlation coefficient between region *i* and region *j*, and *N* is the number of regions.

Similar to the functional maps, individual GM maps were also partitioned into 116 ROIs and the regional mean GM values were extracted as features for that subject.

Finally, for each subject, three functional maps (ALFF, ReHo and RFCS) and one structural map (GM) were obtained, and for each map, we extracted 116 features from the 116 AAL ROIs. For a given ROI, ALFF, ReHo and RFCS reflect the degree of regional activity, the degree of regional synchronization and the degree of global synchronization of spontaneous neuronal activity, respectively; and the GM reflects the morphometric characteristics. Therefore, for each subject, we had 116 × 4 features which convey different types of information.

### Feature selection

The dimension of original features is much higher than the number of samples, which might lead to the curse of dimensionality problem and high complexity. To speed up computation and to improve the classifier performance [44], a feature selection step was adopted to remove irrelevant or redundant features. Two-sample t-tests were performed to determine features that showed differences between MWoA and HC groups. Only features with a *p* value smaller than the predefined threshold (*p*<0.05, uncorrected) were retained. This process was performed independently for each feature, ignoring the relationship (redundant or complementary) with other features. To jointly consider the discriminative power among features, we employed the SVM-recursive feature elimination (SVM-RFE) method [45] for further feature selection. SVM-RFE is a backward selection technique that iteratively removes as many non-informative features as possible while retains features that carry discriminative information. The above mentioned feature selection scheme was performed separately on each feature type. Of note, all the procedures of feature selection were constrained on the training set of each leave-one-out cross-validation (LOOCV) fold.

### Multi-kernel SVM

In order to effectively integrate different feature vectors, a multi-kernel SVM [46] was used. We constructed a base kernel for each feature level, and then mixed these base kernels by a weighted linear combination. Let *F* be the number of the base kernels, then the final kernel can be expressed as

$$K(x_{i},x)=\sum_{f=1}^{F}\beta_{f}k^{\left(f\right)}(x_{i}^{\left(f\right)},x^{\left(f\right)})$$ (2)

where **x**_*i*_ is the feature vector of the *i*-th training sample; **x** is the feature vector of the test sample; *k*^(*f*)^ (**x** (*f*) *i*, **x** ^(*f*)^) is the *f*-th kernel function; and *β*_*f*_ ≥ 0 is the weighting factor of *f-*th kernel function with the constraint of ∑ *fβ*_*f*_ = 1.

The final kernel matrix can be naturally embedded into the conventional single-kernel SVM. Applying a linear SVM to the final kernel, the decision function for the predicted label can be obtained as below:

$$F(x)=sign\left(\sum_{i=1}^{N}y_{i}\alpha_{i}K(x_{i},x)+b\right)$$ (3)

where *N* is the number of training samples, *y*_*i*_∈{-1, +1} is the corresponding class label, *α*_*i*_ is the Lagrangian multiplier, and *b* is a bias.

**Fig 1:**
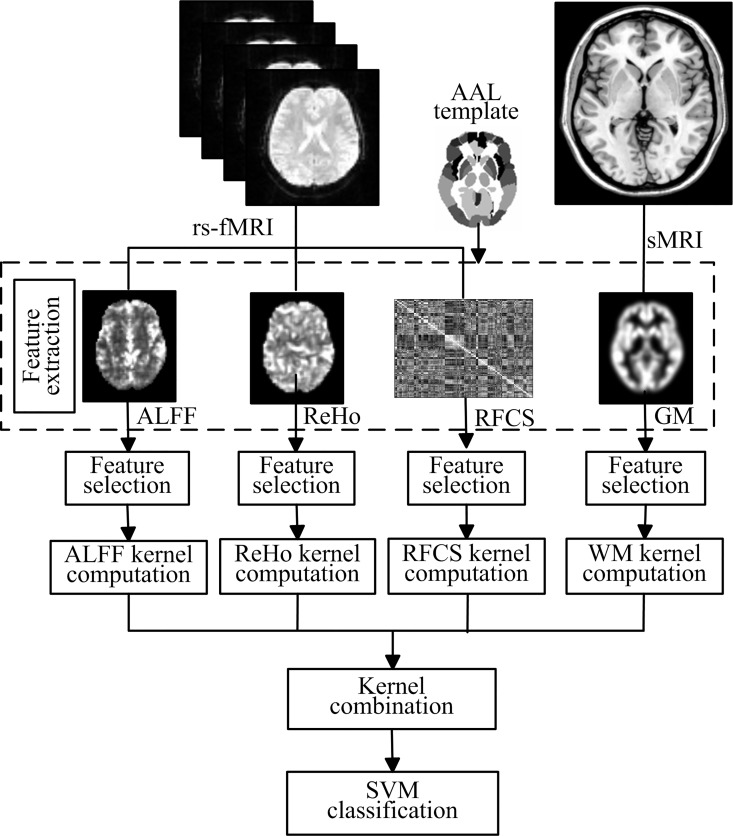
Schematic illustration of the multi-feature combination and classification. ALFF, ReHo, RFCS and GM measures are used to map resting-state brain function and brain structure, respectively. A SVM classifier is then designed using a multi-kernel combination strategy to classify MWoA and HC.

The weights of different kernels in the multi-kernel SVM are learned based on the training samples. The reduced gradient method that converges rapidly and efficiently is chosen to optimize the kernel weights and SVM classifier. The optimization procedure is iterative: given the current solution of kernel weights, it solves a classical SVM with the combined kernel; then updates the kernel weights. This two-step process is repeated until the Armijo’s rule [47] is met. As explained above, the multi-kernel SVM can provide a convenient and effective way for fusing various features from different modalities. In our case, we focused on multiple features from two modalities: rs-fMRI and sMRI. Fig 1 gives the schematic illustration of our multi-feature combination and classification method.

### Cross-validation

Cross-validation is often used to assess the generalizability of a model and to ensure that the model is not overfitted. Here, we used the LOOCV strategy to estimate the performance of the proposed framework. In detail, one sample was designated as a test sample, and the remaining samples were used to train the classifier. For each feature *a*_*i*_ in the training samples, a common feature normalization scheme was adopted: *a*_*i*_ = (*a*_*i*_-*ā*_*i*_)/*σ*_*i*_, where *ā*_*i*_ and *σ*_*i*_ are the mean and standard deviation of the *i*-th feature across all training samples, respectively. The estimated *ā*_*i*_ and *σ*_*i*_ would be used to normalize the corresponding feature of the test sample. In the LOOCV procedure, features used for classification were chosen from the normalized training samples. Specifically, after the filter-based feature selection (t-test), the retained features were further selected by using the SVM-RFE approach, in which an SVM classifier was repeatedly trained; and at each iteration, the square of the weight vector coefficient was used as the ranking criterion to remove the lowest ranking feature. SVM-RFE allowed us to derive an accuracy measure for each feature elimination level from which we determined the minimum number of features required to produce equivalent accuracy to all features. Then the determined features were used to train the multi-kernel SVM classifier. Optimal kernel weight *β*_*f*_ and optimal multi-kernel SVM model were obtained and applied to the test sample. The whole process were repeated until all samples have been left out for test. The final accuracy was computed by averaging the accuracies from all tests. Accuracy, sensitivity and specificity were defined based on the prediction results of LOOCV, to quantify the performance of all compared methods:

$$\text{Sensitivity}=\frac{TP}{TP+FN}$$ (4)

$$\text{Specificity}=\frac{TN}{TN+FP}$$ (5)

$$\text{Accuracy}=\frac{TP+TN}{TP+FN+TN+FP}$$ (6)

where *TP* denotes the number of patients correctly classified; *FN* denotes the number of patients classified as controls; *TN* denotes the number of controls correctly predicted; and *FP* denotes the number of controls classified as patients. We also calculated the area under the receiver operating characteristic curve (AUC) to illustrate the performance of classification.

## Results

### Comparison of classification performance

In classifications based on different feature types, the same feature extraction and selection criteria were used. The generalizability of these classifiers was estimated by using the LOOCV approach. We adopted traditional single-kernel SVM classifier for single-type feature classification, and multi-kernel SVM classifier for multi-type features classification. All the SVM classifiers were implemented with the linear kernel and the default penalty parameter *C* = 1. In case of direct feature concatenation, we linked the 116 × 4 features (ALFF, ReHo, RFCS and GM) into a long feature vector, and used the traditional single-kernel SVM to perform the classification. We also applied the M3 method in which features are trained using a multi-classifier based on four maximum uncertainty linear discriminate analysis base classifiers [32]. The proposed framework obtained a classification accuracy of 83.67%, with a sensitivity of 92.86% and a specificity of 71.43%, which were better than the results of any single-type feature or other multi-type feature combinations. The classification performance of all the feature types were summarized in Table 2, and the top 10 features most frequently selected in the proposed method were listed in Table 3.

**Table 2:** Classification performance using different types of feature.

Feature types	ACC (%)	SEN (%)	SPE (%)	AUC
ALFF	65.31	85.71	38.10	0.69
ReHo	67.35	71.43	61.90	0.67
RFCS	63.27	82.14	38.10	0.68
GM	71.43	85.71	52.38	0.83
ALFF+ReHo	69.39	82.14	52.38	0.70
ALFF+RFCS	64.58	85.71	33.33	0.54
ALFF+GM	70.83	89.29	42.86	0.74
ReHo+GM	72.92	85.71	52.38	0.75
ReHo+RFCS	71.43	82.14	57.14	0.75
RFCS+GM	75.00	92.86	47.62	0.78
ALFF+ReHo+RFCS	72.92	85.71	52.38	0.75
ALFF+ReHo+GM	75.51	89.29	57.14	0.78
ALFF+RFCS+GM	79.59	89.29	66.67	0.84
ReHo+RFCS+GM	73.47	85.71	57.14	0.71
Concatenation	67.35	78.57	52.38	0.74
M3 method	73.47	66.67	78.57	0.82
Proposed	83.67	92.86	71.43	0.83

SEN = sensitivity, SPE = specificity, ACC = accuracy, AUC = area under receive operating characteristic curve. “+” indicates combination of the given types of features; “Concatenation” means all four types of feature were concatenated into a long feature vector.

**Table 3:** Top 10 frequently selected features for proposed classification.

Feature	Regions	Count
ALFF	Left anterior cingulate gyrus	41
	Left posterior cingulate gyrus	39
	Left lenticular nucleus, pallidum	25
	Left inferior frontal gyrus, opercular part	13
	Right superior temporal gyrus	11
	Right inferior frontal gyrus, opercular part	10
	Right posterior cingulate gyrus	10
	Vermis_1&2	9
	Right inferior parietal lobule	6
	Right cerebelum_Crus1	5
ReHo	Right inferior parietal lobule	37
	Right superior temporal gyrus	36
	Left lenticular nucleus, putamen	31
	Left cuneus	27
	Right insula	21
	Left lenticular nucleus, pallidum	20
	Right hippocampus	6
	Right Cerebelum_9	6
	Left superior frontal gyrus, medial orbital	5
	Right lenticular nucleus, putamen	5
RFCS	Left superior frontal gyrus, orbital part	41
	Left amygdala	39
	Right amygdala	39
	Left hippocampus	24
	Right Cerebelum_Crus2	18
	Right inferior frontal gyrus, triangular part	15
	Right Cerebelum_9	12
	Right superior temporal gyrus	11
	Right Cerebelum_7	11
	Vermis_10	4
GM	Left supplementary motor area	39
	Left hippocampus	38
	Right parahippocampal gyrus	33
	Left parahippocampal gyrus	17
	Right hippocampus	9
	Left precentral gyrus	5
	Right precentral gyrus	5
	Left superior frontal gyrus	5
	Right superior frontal gyrus	4
	Right inferior frontal gyrus, opercular part	4

**Fig 2:**
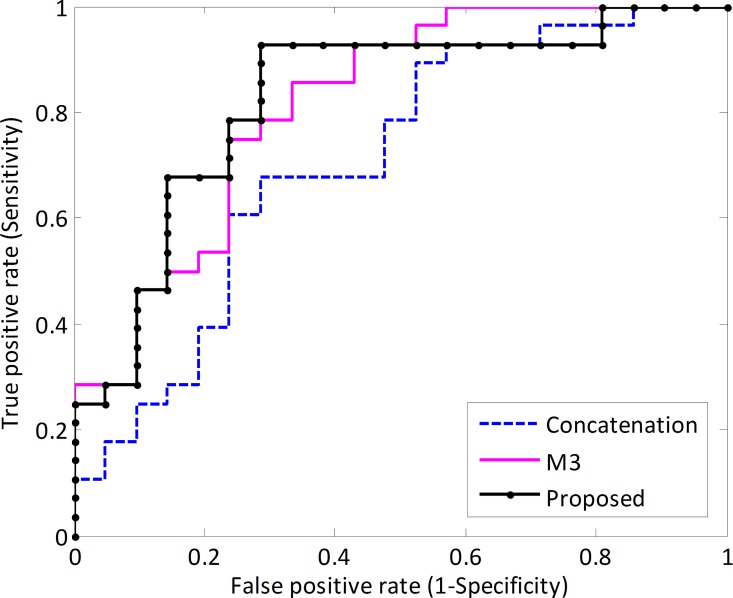
Classification performance of the proposed framework. ROC curve of the classifier, showing the trade-off between sensitivity (y-axis) and specificity (x-axis, 1-specificity). The area under the ROC curve is 0.83 for the proposed approach.

In the last step of the framework, discriminative score of each test subject was acquired by the SVM classifier and then the sign of these scores were used for classification (e.g., positive indicates HC and negative indicates MWoA). The threshold for classification is chosen to be 0 for efficiency, but in order to evaluate the performance of the classifier this threshold can be varied across the range of all possible values obtained. With varying thresholds, the receiver operating characteristic (ROC) curve was plotted (Fig 2). The larger the area under ROC obtained, the better the classification performance achieved. AUC of the proposed framework was 0.83, indicating an excellent discrimination power.

### The most discriminative features

**Fig 3:**
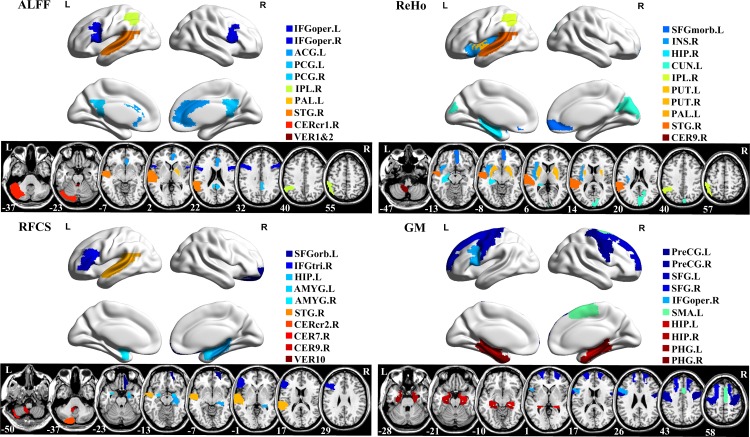
Top ten most discriminative features (regional ALFF, ReHo, RFCS and GM). To visually represent the relative contribution of brain regions for classification, the ROIs were projected onto the cortical surface (top) and shown in 2D slice images (down). Different colors in the figure indicate different brain regions. The surface maps were visualized using BrainNet Viewer (http://www.nitrc.org/projects/bnv/) and the 2D slice map was generated using MRIcron (http://www.mccauslandcenter.sc.edu/mricro/mricron/). L: left, R: right.

Since the feature selection in each fold was performed based on the training set, the selected features slightly differ across different cross-validation folds. Therefore, we defined the features that were frequently selected in all cross-validations as the most discriminative features. The brain regions from where the top ten ALFF, ReHo, RFCS and GM features were selected are provided in Fig 3. Based on the selected ALFF feature, the ten most discriminative regions were the bilateral inferior frontal gyrus, left anterior cingulate gyrus, bilateral posterior cingulate gyrus, right inferior parietal lobule, left lenticular nucleus, right superior temporal gyrus, right cerebellum and vermis. For ReHo, the regions with relative good classification power included the left superior frontal gyrus, right insula, right hippocampus, left cuneus, right inferior parietal lobule, bilateral lenticular nucleus, right superior temporal gyrus, and right cerebellum. The most discriminative regions for RFCS included the left superior frontal gyrus, right inferior frontal gyrus, left hippocampus, bilateral amygdale, right superior temporal gyrus, right cerebellum and vermis. GM regions with relative high classification power included the bilateral precentral gyrus, bilateral superior frontal gyrus, right inferior frontal gyrus, left supplementary motor area, bilateral hippocampus, and the bilateral parahippocampal gyrus. For all feature types, the mostly selected regions are the anterior cingulate cortex, prefrontal cortex, orbitofrontal cortex, and the insula.

## Discussion

To our best knowledge, this is the first study to demonstrate the advantage of multi-type features (sMRI and rsfMRI) integration over single feature approach in the discrimination between MWoA and HC. In general, the more feature types we included, the better performance we obtained. In contrast to the best classification achieved by single-type feature (GM), our framework achieved a higher classification accuracy (83.64% vs. 71.43%). This validated our hypothesis that the combination of different types of features should integrate more effective information into the SVM kernel, since they reflect complementary pathological aspects of diseases. It is worth noting that identifying disease by combining biomarkers from different feature types with different data fusion methods is still an open area for research.

In the classification using multi-modal imaging data, the feature combining method is a key point for effective information integration. The simple and common practice is concatenating all features into a longer feature vector. However, in this study the direct concatenation only achieved an accuracy of 67.35%, which is even lower than the result of the single GM feature, indicating that it’s not an effective combination method. Rather, when we combined features from different modalities using the multi-kernel combination strategy, which firstly combined the kernel matrices of different features into a mixed kernel matrix, and then using it to train a single SVM model, much better classification performance was achieved. This strategy assigns kernel weights to different feature types, which may partially interpret the improved capability in integrating comprehensive and complementary information for the purpose of identification. Our framework also performed better in comparison with the M3 method, which used multi-classifiers to integrate multi-modal information, though better specificity was achieved by M3. This may implicate the advantageous capability of M3 method to reduce the occurrence of misdiagnosis in some circumstance.

Feature selection is a useful and important process to remove irrelevant or redundant features for dimensionality reduction and improving the performance of the classifier. The filter-based (t-test) and wrapper-based (SVM-RFE) feature selection methods were used in this work. We would point out that we were not using SVM-RFE to optimize predictive accuracy but to remove non-informative data from the extracted features and to find the most parsimonious feature representation. Here, the behavior of predictive accuracy was evaluated as a function of the number of features in the data. The SVM-RFE algorithm iteratively removes non-informative features from the data set and derives an accuracy measure for each feature elimination level. In this way the minimum number of features required to produce equivalent accuracy to all features can be obtained.

Among all our single-type feature classifications, the highest accuracy was obtained when the GM features were used; as well, in all the two-type and three-type feature combinations, the ones that incorporated GM features always performed relatively better. In a recent study by Schwedt et al. [11], three structural features (regional cortical thickness, cortical surface area, and volume) were used for migraine identification and achieved a desirable classification accuracy. These results collectively suggest that structural information might be essential for migraine identification when using a machine learning approach. Considering other machine learning researches into Alzheimer’s disease [46] and Parkinson’s disease [48], which are all based on structural imaging features, we would agree with the argument that structural imaging markers may have greater weight in the diagnostic and prognostic judgment for neurological diseases [49].

Our proposed framework sought to identify the most discriminative features between MWoA patients and healthy controls. The brain regions with top discriminative powers were partially overlap with those reported in the previous MWoA studies that applied conventional univariate statistical analysis to functional and structural imaging data. For example, altered ALFF has been identified in the anterior cingulate gyrus and inferior frontal gyrus [12]; ReHo changes have been revealed in the superior frontal gyrus [13], insula, superior temporal gyrus, lenticular nucleus, cerebellum, hippocampus, cuneus and the inferior parietal lobule [14]; and, abnormal functional connectivity has been reported in the prefrontal and temporal regions [16, 18], as well as amygdala and visceroceptive cortex [17]. Regions showing high discriminative power for GM features in the current study, such as the precentral gyrus, superior frontal gyrus, inferior frontal gyrus and supplementary motor area, were also reported with deficits in previous voxel based morphometric studies of MWoA [9, 10, 16]. But with one important note, the regions shown on discrimination maps only indicate their relatively high contributions to the classification, but are not equivalent to those identified from mass-univariate analyses that are of more pathophysiology relevance.

Our study should be still taken as a preliminary proof-of-concept study. It proposes a promising approach for the future translation of neuroimaging into patient benefit. The pipeline we used includes the preprocessing of sMRI and rs-fMRI data using standard analytical software (SPM and DPARSF); extraction of feature vectors that consist of regional ALFF, ReHo, RFCS and GM values; selection of the extracted features; and application of the multi-kernel SVM to the selected feature vector. Once the multi-kernel SVM classifier is trained and a decision function is generated, a new sample could be classified in minutes. Although our approach requires replication and validation in larger samples, it provides initial evidence of a rapid and accessible methodology that could potentially aid clinical decisions.

One imperfection of this study is that in the depression and anxiety assessments the MWoA group scored significantly higher than healthy controls. Clinically diagnosed depression and anxiety are reported to be associated with brain alterations that overlap with the observations in the current study; for example, the ALFF changes in the cerebellum, anterior cingulate cortex and inferior frontal gyrus in depression [27], and fronto-amygdalar functional connectivity changes in anxiety [50]. We acknowledge that this may raise concerns about the bias introduced into the feature pattern, however, the patients’ HAMD and HAMA scores are still far below the diagnostic threshold for depression and anxiety disorder, so the differences are more likely to be emotional fluctuations caused by migraine. The effects of these differences on imaging data, if any, should be minor. Of course, an ideal research should only recruit MWoA but without any other combined symptoms.

Other possible limitations of our study should also be noted. Firstly, we only included rs-fMRI and sMRI for multi-modal classification. Other data modalities e.g., task fMRI, diffusion MRI and electroencephalogram, which all provide additional information, should be considered in future studies. Secondly, we used the AAL atlas to parcellate the whole brain for ROI definition. Though this method simplified computation, recent studies did find that different parcellation schemes could affect brain network analysis [51–53]. Thus, it’s needed to apply our method to other brain atlases, or even at voxel level, to investigate the impact of brain parcellation on classification. Finally, considering the relative small sample size (49 subjects in total), the classifier is only specific to the current dataset. In the future, we would like to use a larger dataset to determine the generalizability of this framework.

## Conclusions

This study proposed a novel framework to discriminate MWoA and HC using ALFF, ReHo, RFCS and GM features derived from rs-fMRI and sMRI scans. Compared with the single-, two-, and three-type feature based classification, the classification performance was improved by integrating all four types of features via a multi-kernel SVM. The promising classification results suggest multi-modal imaging data based pattern-classification as a direction that deserves more comprehensive investigations for MWoA discrimination.

## Supporting Information

S1 Data Discriminative scores for the classifier. Relevant data underlying the findings described in the manuscript.
(DOC)

S2 Data Features retained for classification in all cross-validation. Relevant data underlying the findings described in the manuscript.
(DOC)

S1 Table Regions of interest of the AAL atlas. (DOC)

S2 Table The number of features retained in the proposed approach per fold. (DOC)
