# Supplementary material for: Discriminative Analysis of Migraine without Aura: Using Functional and Structural MRI with a Multi-Feature Classification Approach
Source: PLoS One. 2016 Sep 30;11(9):e0163875. doi: 10.1371/journal.pone.0163875 (PMC5045214; doi:10.1371/journal.pone.0163875)
Supplement: S1 Data — Relevant data underlying the findings described in the manuscript. (DOC) [file pone.0163875.s001.doc]

**S1 Data. Discriminative scores for the classifier. Relevant data underlying the findings described in the manuscript.**

| **Example No.** | **True class** | **Classifier score** |
| --- | --- | --- |
| 1 | P | 0.9562 |
| 2 | P | 0.8935 |
| 3 | P | 0.8249 |
| 4 | P | 0.7243 |
| 5 | P | 0.6919 |
| 6 | P | 0.6661 |
| 7 | P | 0.5907 |
| 8 | N | 0.5431 |
| 9 | P | 0.5347 |
| 10 | N | 0.5127 |
| 11 | P | 0.5097 |
| 12 | P | 0.4893 |
| 13 | P | 0.4752 |
| 14 | P | 0.4575 |
| 15 | P | 0.4399 |
| 16 | N | 0.4332 |
| 17 | P | 0.4082 |
| 18 | P | 0.3785 |
| 19 | P | 0.3577 |
| 20 | P | 0.3428 |
| 21 | P | 0.2995 |
| 22 | P | 0.2787 |
| 23 | N | 0.2714 |
| 24 | N | 0.2491 |
| 25 | P | 0.1676 |
| 26 | P | 0.1497 |
| 27 | P | 0.1018 |
| 28 | N | 0.0965 |
| 29 | P | 0.096 |
| 30 | P | 0.0429 |
| 31 | P | 0.0415 |
| 32 | P | 0.0367 |
| 33 | N | -0.004 |
| 34 | N | -0.0131 |
| 35 | N | -0.0635 |
| 36 | N | -0.0709 |
| 37 | N | -0.2017 |
| 38 | N | -0.2057 |
| 39 | N | -0.2135 |
| 40 | N | -0.3202 |
| 41 | N | -0.3256 |
| 42 | N | -0.3673 |
| 43 | N | -0.3916 |
| 44 | P | -0.4215 |
| 45 | P | -0.4238 |
| 46 | N | -0.4704 |
| 47 | N | -0.625 |
| 48 | N | -0.6527 |
| 49 | N | -1.2092 |
