# Supplementary material for: Discriminative Analysis of Migraine without Aura: Using Functional and Structural MRI with a Multi-Feature Classification Approach
Source: PLoS One. 2016 Sep 30;11(9):e0163875. doi: 10.1371/journal.pone.0163875 (PMC5045214; doi:10.1371/journal.pone.0163875)
Supplement: S2 Data — Relevant data underlying the findings described in the manuscript. (DOC) [file pone.0163875.s002.doc]

**S2 Data. Features retained for classification in all cross-validation. Relevant data underlying the findings described in the manuscript.**

| **No.** | **ALFF_retained** | **ReHo_retained** | **RFCS_retained** | **GM_retained** |
| --- | --- | --- | --- | --- |
| 1 | [31;62;75] | [38;73;82] | [5;14;82] | [19;37] |
| 2 | [11;31;35] | [66;73;75;82] | [5;14;82;94;106] | [19;37;39;40] |
| 3 | [31;35;36;75;92] | [30;45;59;82;115] | [5;41;42;94;102;106] | [1;2;3;4;12;38;39;86] |
| 4 | [12;31;36;62;75] | [28;73;75;82] | [5;37;41;42;94;106] | [19;37;39;40] |
| 5 | [11;31;36;92;104] | [27;28;30;45;104;108] | [5;41;82;102;106] | [19;40] |
| 6 | [31;35;75;109] | [28;62;73;74;75;76;82;106] | [41;82;106] | [3;4;37;38;39;40] |
| 7 | [12;62;75] | [30;66;73;74;75] | [37;41;82;94;102;106] | [19;37] |
| 8 | [31;35;75;109] | [73;82] | [14;37;42;94;102;106] | [19;40] |
| 9 | [31;35;109;115] | [30;45;62;75;82] | [5;41;42] | [19;37;40] |
| 10 | [35;75] | [30;45;62;75;82] | [5;14;37;41;82;106] | [19;37;39;40] |
| 11 | [12;31;35;75] | [73;75] | [5;37;40;82;106] | [19;37;39;40] |
| 12 | [31;35;75] | [30;45;75;82] | [5;37;41;42;116] | [19;37;40] |
| 13 | [12;31;82] | [61;62;73;75;82] | [5;14;28;37;82;106] | [19;37;40] |
| 14 | [31;35] | [62;73;82] | [5;41;42;94] | [19;40] |
| 15 | [11;31;35;36;75;92] | [30;45;59;62;104] | [14;37;41;42] | [1;2;3;4;12;38;39;86] |
| 16 | [31;62;75;82] | [62;73;75;82] | [5;41;42;82;94] | [19;37;40] |
| 17 | [31;62] | [38;62;73] | [5;14;37;41;42] | [2;31;37;39;40] |
| 18 | [31;35;109] | [25;45;62;73;82] | [5;14;41;42] | [19;37;40] |
| 19 | [31;35;36;82;109] | [25;30;59;60;62;73;75;82] | [5;37;41;42] | 19 |
| 20 | [35;109] | [30;45;62;82] | [5;14;37;41;42;94] | [19;37;62] |
| 21 | [11;35;62;75;82] | [30;45;62;82] | [5;37;41;42;106] | [2;12;19;37;40] |
| 22 | [11;12;31;35;75] | [25;45;62;73;82] | [5;14;37;41;42;94] | [19;37] |
| 23 | [12;31;36;75] | [30;45;62;75;82] | [5;37;41;42;94] | [1;3;4;19;20;38;39;40] |
| 24 | [31;75;77] | [65;73;75] | [5;41;42] | [1;2;3;12;38;39;86] |
| 25 | [31;35;75] | [30;38;62;73;82;104] | [5;41;42;94;102] | [19;37;39;40] |
| 26 | [11;31;35;36;75] | [45;62;73;82] | [5;41;82] | [19;37;40] |
| 27 | [12;31;35] | [45;62;73;82] | [5;41;42;116] | [19;37] |
| 28 | [31;35;75;82] | [25;73;82] | [5;42;116] | [19;37] |
| 29 | [31;35;75] | [30;45;62;82] | [14;37;42;94;102;115] | [1;19;37;38;39;40] |
| 30 | [31;35;92] | [38;62;73;74;75;104] | [5;41;42] | [19;37;39;40] |
| 31 | [35;82] | [62;73;82] | [5;37;41;42] | [19;37;40] |
| 32 | [31;35;36] | [30;45;62;75;104;109] | [5;14;41;42;94] | [19;37;38;40] |
| 33 | [11;31;77] | [38;62;73] | [5;37;41;42] | [19;37;40] |
| 34 | [31;35] | [45;62;73;82] | [5;37;42] | [19;37] |
| 35 | [31;35;75] | [45;62;73] | [5;14;41;42] | [33;37;38;39;40] |
| 36 | [31;35;75] | [45;62;73;75;82] | [5;14;40;41;42] | [19;37] |
| 37 | [31;35;75] | [30;45;62;74;75;82] | [5;41;42;94;102] | [33;37] |
| 38 | [11;31;35;92] | [25;62;73;74;104] | [5;41;42;102;116] | 19 |
| 39 | [31;35;36;82] | [45;62;73;82] | [5;37;41;42] | [29;37;40] |
| 40 | [35;109] | [30;45;62;82] | [5;37;41;42;94] | [19;37;39] |
| 41 | [31;35;109] | [62;65;66] | [5;37;41;42;62] | [19;37;38;40] |
| 42 | [11;12;31;35] | [62;66;82] | [28;37;42;94;102] | [30;37;40] |
| 43 | [31;35;82] | [30;45;62;82] | [5;41;42] | [19;37;39;40] |
| 44 | [31;35;109] | [30;45;62;73;82;115] | [5;41;42] | [19;40] |
| 45 | [11;35;82] | [30;62;65;73;82;116] | [5;14;37;41;42;106] | [19;37] |
| 46 | [11;12;31;35] | [45;62;73] | [37;82;94;102] | [19;37;40] |
| 47 | [11;31;35;36;75;116] | [30;45;62;75;76;82] | [5;41;42] | [19;40] |
| 48 | [31;35;75;82] | [45;62;73;75;82] | [5;28;41;42;102] | [19;37;40] |
| 49 | [11;12;35;75;82] | [30;38;45;75;82] | [41;42;94] | [29;37;39;40] |
