# Supplementary material for: Discriminative Analysis of Migraine without Aura: Using Functional and Structural MRI with a Multi-Feature Classification Approach
Source: PLoS One. 2016 Sep 30;11(9):e0163875. doi: 10.1371/journal.pone.0163875 (PMC5045214; doi:10.1371/journal.pone.0163875)
Supplement: S1 Table — (DOC) [file pone.0163875.s003.doc]

**S1 Table.** Regions of interest of the AAL atlas.

| **No.** | **Regions** | **Abbr.** | **No.** | **Regions** | **Abbr.** |
| --- | --- | --- | --- | --- | --- |
| (1,2) | Precentral gyrus | PreCG | (63,64) | Supramarginal gyrus | SMG |
| (3,4) | Superior frontal gyrus | SFG | (65,66) | Angular gyrus | ANG |
| (5,6) | Superior frontal gyrus, orbital part | SFGorb | (67,68) | Precuneus | PCUN |
| (7,8) | Middle frontal gyrus | MFG | (69,70) | Paracentral lobule | PCL |
| (9,10) | Middle frontal gyrus, orbital part | MFGorb | (71,72) | Caudate nucleus | CAU |
| (11,12) | Inferior frontal gyrus, opercular part | IFGoper | (73,74) | Lenticular nucleus, putamen | PUT |
| (13,14) | Inferior frontal gyrus, triangular part | IFGtri | (75,76) | Lenticular nucleus, pallidum | PAL |
| (15,16) | Inferior frontal gyrus, orbital part | IFGorb | (77,78) | Thalamus | THA |
| (17,18) | Rolandic operculum | ROL | (79,80) | Heschl gyrus | HES |
| (19,20) | Supplementary motor area | SMA | (81,82) | Superior temporal gyrus | STG |
| (21,22) | Olfactory cortex | OLF | (83,84) | Temporal pole: superior temporal gyrus | TPOsup |
| (23,24) | Superior frontal gyrus, medial | SFGmed | (85,86) | Middle temporal gyrus | MTG |
| (25,26) | Superior frontal gyrus, medial orbital | SFGmorb | (87,88) | Temporal pole: middle temporal gyrus | TPOmid |
| (27,28) | Gyrus rectus | REC | (89,90) | Inferior temporal gyrus | ITG |
| (29,30) | Insula | INS | (91,92) | Cerebelum_Crus1 | CERcr1 |
| (31,32) | Anterior cingulate gyrus | ACG | (93,94) | Cerebelum_Crus2 | CERcr2 |
| (33,34) | Median cingulate gyrus | MCG | (95,96) | Cerebelum_3 | CER3 |
| (35,36) | Posterior cingulate gyrus | PCG | (97,98) | Cerebelum_4&5 | CER4&5 |
| (37,38) | Hippocampus | HIP | (99,100) | Cerebelum_6 | CER6 |
| (39,40) | Parahippocampal gyrus | PHG | (101,102) | Cerebelum_7 | CER7 |
| (41,42) | Amygdala | AMYG | (103,104) | Cerebelum_8 | CER8 |
| (43,44) | Calcarine fissure | CAL | (105,106) | Cerebelum_9 | CER9 |
| (45,46) | Cuneus | CUN | (107,108) | Cerebelum_10 | CER10 |
| (47,48) | Lingual gyrus | LING | 109 | Vermis_1&2 | VER1&2 |
| (49,50) | Superior occipital gyrus | SOG | 110 | Vermis_3 | VER3 |
| (51,52) | Middle occipital gyrus | MOG | 111 | Vermis_4&5 | VER4&5 |
| (53,54) | Inferior occipital gyrus | IOG | 112 | Vermis_6 | VER6 |
| (55,56) | Fusiform gyrus | FFG | 113 | Vermis_7 | VER7 |
| (57,58) | Postcentral gyrus | PoCG | 114 | Vermis_8 | VER8 |
| (59,60) | Superior parietal gyrus | SPG | 115 | Vermis_9 | VER9 |
| (61,62) | Inferior parietal lobule | IPL | 116 | Vermis_10 | VER10 |

Odd and even numbers (1-108) represent brain regions of left and right hemispheres, respectively.
