# Supplementary material for: Discriminative Analysis of Migraine without Aura: Using Functional and Structural MRI with a Multi-Feature Classification Approach
Source: PLoS One. 2016 Sep 30;11(9):e0163875. doi: 10.1371/journal.pone.0163875 (PMC5045214; doi:10.1371/journal.pone.0163875)
Supplement: S2 Table — (DOC) [file pone.0163875.s004.doc]

**S2 Table. The number of features retained in the proposed approach per fold**.

| **Fold** | **ALFF** | **ReHo** | **RFCS** | **GM** | **Fold** | **ALFF** | **ReHo** | **RFCS** | **GM** |
| --- | --- | --- | --- | --- | --- | --- | --- | --- | --- |
| **1** | 3 | 3 | 3 | 2 | **26** | 5 | 4 | 3 | 3 |
| **2** | 3 | 4 | 5 | 4 | **27** | 3 | 4 | 4 | 2 |
| **3** | 5 | 5 | 6 | 8 | **28** | 4 | 3 | 3 | 2 |
| **4** | 5 | 4 | 6 | 4 | **29** | 3 | 4 | 6 | 6 |
| **5** | 5 | 6 | 5 | 2 | **30** | 3 | 6 | 3 | 4 |
| **6** | 4 | 8 | 3 | 5 | **31** | 2 | 3 | 4 | 3 |
| **7** | 3 | 5 | 6 | 2 | **32** | 3 | 6 | 5 | 4 |
| **8** | 4 | 2 | 6 | 2 | **33** | 3 | 3 | 4 | 3 |
| **9** | 4 | 5 | 3 | 3 | **34** | 2 | 4 | 3 | 2 |
| **10** | 2 | 5 | 6 | 4 | **35** | 3 | 3 | 4 | 5 |
| **11** | 4 | 2 | 5 | 4 | **36** | 3 | 5 | 5 | 2 |
| **12** | 3 | 4 | 5 | 3 | **37** | 3 | 6 | 5 | 2 |
| **13** | 3 | 5 | 6 | 3 | **38** | 4 | 5 | 5 | 1 |
| **14** | 2 | 3 | 4 | 2 | **39** | 4 | 4 | 4 | 3 |
| **15** | 6 | 5 | 4 | 8 | **40** | 2 | 4 | 5 | 3 |
| **16** | 4 | 4 | 5 | 3 | **41** | 3 | 3 | 5 | 4 |
| **17** | 2 | 3 | 5 | 5 | **42** | 4 | 3 | 5 | 3 |
| **18** | 3 | 5 | 4 | 3 | **43** | 3 | 4 | 3 | 4 |
| **19** | 5 | 8 | 4 | 1 | **44** | 3 | 6 | 3 | 2 |
| **20** | 2 | 4 | 6 | 3 | **45** | 3 | 6 | 6 | 2 |
| **21** | 5 | 4 | 5 | 5 | **46** | 4 | 3 | 4 | 3 |
| **22** | 5 | 5 | 6 | 2 | **47** | 6 | 6 | 3 | 2 |
| **23** | 4 | 5 | 5 | 8 | **48** | 4 | 5 | 5 | 3 |
| **24** | 3 | 3 | 3 | 7 | **49** | 5 | 5 | 3 | 4 |
| **25** | 3 | 6 | 5 | 4 |  |  |  |  |  |
